# The Mismatch Negativity: An Indicator of Perception of Regularities in Music

**DOI:** 10.1155/2015/469508

**Published:** 2015-10-04

**Authors:** Xide Yu, Tao Liu, Dingguo Gao

**Affiliations:** Department of Psychology, Sun Yat-Sen University, No. 135 Xingang Xi Road, Guangzhou 510275, China

## Abstract

This paper reviews music research using Mismatch Negativity (MMN). MMN is a deviation-specific component of auditory event-related potential (EPR), which detects a deviation between a sound and an internal representation (e.g., *memory trace*). Recent studies have expanded the notion and the paradigms of MMN to higher-order music processing such as those involving short melodies, harmony chord, and music syntax. In this vein, we firstly reviewed the evolution of MMN from sound to music and then mainly compared the differences of MMN features between musicians and nonmusicians, followed by the discussion of the potential roles of the training effect and the natural exposure in MMN. Since MMN can serve as an index of neural plasticity, it thus can be widely used in clinical and other applied areas, such as detecting music preference in newborns or assessing wholeness of central auditory system of hearing illness. Finally, we pointed out some open questions and further directions. Current music perception research using MMN has mainly focused on relatively low hierarchical structure of music perception. To fully understand the neural substrates underlying processing of regularities in music, it is important and beneficial to combine MMN with other experimental paradigms such as early right-anterior negativity (ERAN).

## 1. Introduction

Music, as an aesthetic entity, is constituted of complex structures and regularities. The basic components of musical sound include pitch, duration, intensity, and timbre. While the high-level elements consist of beat, tempo, chord, rhythm, and harmony melody following certain rules [1, 2]. It has been acknowledged that music plays important roles in evolution and development of human cognitive functions such as intelligence [3], language [4], and memory [5] and in turn affects human social behaviors, for example, emotion management, self-identity, and interpersonal relationship [6]. More recently, growing neurology research has further reported a close relationship between music and rehabilitation/remission of some neural diseases, such as Alzheimer's disease [7–12], Parkinson's disease [13], autism [14], and traumatic brain injury [15]. Therefore, it is important and beneficial to systemically examine the neural substrates underlying music perception/appreciation.

Because of its fundamental importance on human cognition and behavior, music has been attracting increasing attention in fields of neuroscience and neurology, opening a new hotspot of research termed as “music neuroscience” [16–18]. Previous literature on music neuroscience has primarily focused on Mismatch Negative (MMN)—a deviation-specific component of auditory event-related potential (ERP) recorded by electroencephalography (EEG)—in “music” perception. MMN is closely related to deviations between different sound features, such as pitch, timbre, location of sound source, intensity, rhythm, and abstract rule. Specifically, MMN peaks at around 100–200 ms after deviation onset with the amplitude and latency altering depending on deviation magnitude and perceptual discriminability [19].

Concerning the neural substrates of MMN, previous EEG studies [20–22] have revealed that the main source of MMN may localize at the supratemporal plane of the parietal lobes and is close to the primary and secondary auditory cortices, while the additional sources are in proximity to the inferior frontocentral lobes [23, 24]. After being found firstly in 1978 [25], MMN has been detected at a wide variety of studies from single sine tone [26, 27], alternating piano tones [28, 29], pitch discrimination [30], timbre processing [31, 32], rhythm [33], and beat [34] to chord and melody [35]. And recently, MMN in music has greatly broken through the limitations of traditional MMN paradigms in sound perception, even though the nature of MMN in detecting deviations from standards will never change. In context-free experiments, for example, frequency deviation in sequences of pure tones, the standardized internal representation is commonly assumed as an auditory memory, induced by frequent repetition of a standard sound, and is hence termed as* “memory trace.”* In the same vein, in music this may interact with internal representation of rules, cultural preferences, and so forth. Thus, the MMN is involved in neural system that detects the violation of rule-based contexts in memory trace.

Due to the social nature of music, various music styles are different in their cultures [36, 37]. From a perspective of social culture, a large number of previous studies have demonstrated that human brain has a neural template that is derived from our musical culture, allowing for the detection of deviations of culture-based regularities by MMN [38, 39]. Besides MMN, on the other hand, ERP research has also revealed several components related with cultural familiarity in terms of music phrase boundary process, such as a P3-like component and music closure positive shift (CPS) [40, 41]. For instance, in a brain-imaging study, Nan et al. (2008) have reported that the culturally familiar, native (i.e., Western) condition elicited higher activation in the bilateral motor regions, the left planum temporale, and the right ventromedial prefrontal cortex than the nonnative condition, indicating better sensorimotor integration. In contrast, the unfamiliar, nonnative music style (i.e., Chinese) yielded higher activation in a right lateralized network, including the angular gyrus, the middle frontal gyrus, and the posterior insula, indicating increased demands on attentional control and auditory processing [42].

These ERP components and functional activities distinguish between music-related neural regularities. However, they touch little on deviation detection in memory trace (induced via frequently repeated standard musical sounds) that can be monitored by MMN. And there is an interesting question that whether or not culture-related neural regularities play a role in facilitation or interference to MMN. More studies are needed to address this issue.

Although MMN is widely detected in music research, it alone is not enough to fully understand the neural substrates underlying music perception, since MMN only reflects simple deviation from internal memory trace. The early right-anterior negativity (ERAN) is a specific response to harmonic violations, reflecting the processing of music-syntactic information. That is, ERAN involves acoustic information structured according to complex and abstract regularities [43]. Hence, combination of MMN and ERAN may provide us more rich and complete information about music perception.

Previous studies have demonstrated that both MMN and ERAN, having a feature of plasticity, are modulated by musical experience and culture surroundings [43], even in early childhood. Thus, they may facilitate cognitive treatments on individuals with amusia and other auditory-related diseases. To achieve this goal, many questions are needed to be resolved in future music neuroscience research, such as how to use MMN and ERAN to deal with the debates of top-down (determined by the implicit or explicit knowledge of music sound) and down-top (context-driven, a human inborn nature) modulations in music and where is the “music processing sub-module” proposed by Peretz and Coltheart [44]?

Following this logical stream, we will firstly review MMN history from sound to music briefly and discuss its critical functions. Focusing on the feature of neuroplasticity in MMN, we will then primarily introduce different MMN responses between musicians and nonmusicians, its underlying mechanisms, and the practical applications. Finally, we will conclude with the role of MMN in music perception and point out further directions in music neuroscience research, for example, combining MMN and ERAN.

## 2. Brief Review on MMN

### 2.1. MMN in Sound

Research on MMN covers a wide range, from simple sound detection to complex music perception. Accordingly, our brief review on MMN begins with studies using sound stimuli followed by ones using music paradigms. Although MMN exists not only in ERP but also in other forms, such as MMN in MEG [45], MMN in PET [46], and MMN in* f*MRI [47, 48], the later ones are out of range of this review, and we will primarily focus on the MMN in ERP. MMN is originally defined as a* deviation*-*sensitive* component of auditory event-related potential (ERP). However, it is worthy to note that the MMN highly depends on the presence of a short-term* memory trace* in the auditory cortex representing the repetitive aspects of the preceding auditory events, which usually lasts for a few seconds. Thus, single sounds, even deviants, with no former sounds during the last few seconds elicit no MMN but enhance obligatory responses of P1, N1, and P2 [49].

By now, most of conclusions about MMN are induced by the research using an oddball paradigm that consists of two sounds with different auditory features [19]. And follow-up researchers only changed some auditory attributions based on the traditional oddball paradigm in a principle of keeping two variants of one feature (one is for standard, and the other is for deviant). The rationale of the traditional oddball paradigm is the detection of the rare deviant stimulus from the relatively high quantity of standard stimulus. For instance, a sequence of frequent, standard sounds (with a probability of occurrence of 0.8 to form a stable memory trace in the auditory cortex) and infrequent deviant sounds (with a probability of 0.2) is randomly presented.

Interestingly, the strength (i.e., amplitude) and the speed (i.e., latency) of the MMN signal are related with both the size of the deviance (i.e., how the infrequent stimulus deviates from the defined memory trace) and the probability of occurrence of the deviant but are independent of the demands of primary tasks. Increasing deviant probability (i.e., decreasing the occurrence probability of standard stimuli) leads to an attenuation of MMN amplitude, which is thought to be partially due to a weakened standard memory trace. More importantly, this attenuation may also result from conflict between “two memory traces.” That is, increased probability of deviant stimuli may lead to development of their own memory trace, which in turn may inhibit the MMN generation of the standard stimuli.

Concerning effect of task demands, the MMN detects sound deviations even in preattentive condition, where the (in)frequent stimuli are presented while participants are engaging in other sound-irrelevant tasks, such as watching a silent movie with subtitles, playing a computer game, or reading a magazine [50, 51]. These results suggest that MMN is sensitive to auditory deviations implicitly with little effect from the explicit task demands.

Last but not least, previous studies have revealed that MMN is elicited not only by basic auditory features (e.g., sine tone and single instrumental tone) but also by different kinds of abstract changes in auditory stimulation such as language grammar and musical syntax violations (for a review, see [52]). That is, we can record the MMN effect even without the so-called “standard stimuli” [53].

### 2.2. Multifeature MMN: From Sound to Music

Almost before 2004, most of studies on MMN have primarily employed the traditional oddball paradigm or its variants. This paradigm, however, has two main questions: one is that the oddball paradigm requires long-time periods in experiments that is unsuitable to children participants and other participants with some cognitive diseases [38]. The other question relates to ecological validity. That is, the oddball paradigm is oversimplified, and in turn, only one MMN acoustic feature can be examined every time. Although the oddball paradigm is highly appropriate to assess sensory dissonance, understanding of realistic acoustic environments and music that have more complex structures and consist of rich components cannot be achieved by the oddball paradigm itself.

To address this issue, Näätänen et al. (2004) have investigated the MMN in a “multi-feature paradigm.” Compared to the traditional oddball paradigm, the multifeature paradigm enables fast recording of responses to several deviant types in one-stimulus sequence, such as intensity, frequency, duration, stimulus omission, timbre, and pitch contour [26]. In addition, the MMN acquired in the new multifeature paradigm is equal in amplitude to those in the traditional oddball paradigm. Thus, the multifeature paradigm is effective and more appropriate to investigation of the music-related MMN. However, there still exists a question. The multifeatures in Näätänen's paradigm were presented without context and rules that might be different from real music.

### 2.3. MMN in Music

In the original multifeature paradigm by Näätänen et al. in 2004 [26], the deviant attributions consist of frequency, intensity, duration, location, and a silent gap, but, as discussed above, all these five features compose no real music due to lack of context and rules. Recently, Vuust et al. (2011) have demonstrated a new, fast, musical multifeature MMN paradigm, in which 6 types of acoustic changes, relevant to musical processing in different musical genres, are presented in the same sequence (termed as melodic multifeature paradigm) [28]. Specifically, 5 of the 6 musical features are aspects of the musical sound that elicit different MMNs depending on levels of musical expertise: pitch mistuning, intensity, timbre, sound-source location, and rhythm [54–56]. And to create a paradigm that could be used to compare nonmusicians to musicians, as well as musicians with different musical genres, they included a pitch slide, which is typical for improvisational music instead of classical music [57].

In comparison with the traditional multifeature paradigm by Näätänen et al. (2004), the melodic multifeature paradigm has thus shown a greater similarity to real music. The traditional multifeature paradigm in music research is based on a simple musical figure, which is well-known in many genres of Western tonal music: the Alberti bass, an accompaniment that is originally encountered in classical music such as Mozart's sonatas or Beethoven's rondos, and is later adopted with variations in other contemporary musical genres. In contrast, the melodic multifeature paradigm is composed of short melodies and includes deviations of complex spectral and temporal regularities, such as melody, rhythm, key, timbre, tuning, and timing.

In sum, using multifeature MMN paradigms and the variants, researchers have demonstrated that the neural substrates underlying conscious musical experience may involve the following three stages: (1) the encoding and temporal integration of each sound characterized by its specific acoustic and perceptual features (e.g., pitch, duration, and timbre) into brief neural traces, (2) the simultaneous maintenance and integration of the neural traces for acoustic features leading possibly to the memorization of musical motifs, and (3) the modulation of sound perception by the memory of the previous sounds (for a review, see [58]).

### 2.4. MMN Features Specific to Music

#### 2.4.1. From Pitch to Interval or Contour

Taking Western tonal music as an example, it is based on a small subset of 12 pitches in the chromatic equal-tempered scale, where the intervals between consecutive pitches are semitones. And this discrete selection of pitches and their relationships to each other form the most fundamental rules of Western tonal music, resulting in jazz, classical, pop musical genres, and so forth. Human beings can extract the pitch difference (interval) with a sensitivity of 10–30 cents [30]. In musical domain, pitch change (frequency change) within chromatic scale is generally named as “out of tune” or “mistune,” and pitch change within diatonic scale is named as “out of key.” Two different pitches form intervals, including melodic interval (tones played sequentially) and harmonic interval (tones played simultaneously). Sounds presented continuously are characterized not only by the pitch, but also by the contour, the perceived duration, the perceived loudness (which does not necessarily coincide with the physical intensity), the timbre, and so forth. These rules imply that the way, in which those musical features are processed, is another important factor for disclosing the neural mechanisms of music perception, distinguished with the general sound perception.

*(1) Pitch Change as out of Tune*. Previous studies have induced a paradox about preattentive versus attentive processing in pitch deviations using MMN paradigm. For instance, Koelsch et al. (1999) have used the ERP MMN to assess the accuracy of* preattentive* pitch discrimination in expert versus novice violin players [59]. In their study, the standard stimuli consisted of major chords of three sinusoidal tones with a perfect major third and fifth. The deviant stimuli were the same with the standard chords, except that the middle tone of the chords was marginally mistuned. The behavioral data revealed that nonmusicians detected 10% of the deviant chords, whereas violin players detected 80%. Consistent with the behavioral result, the ERP data showed that the deviants elicited the MMN in the musicians, but not in the nonmusicians. Specifically, in the discrimination condition, a significant MMN was followed by N2b and P3b deflections only in musicians. These results suggest that pitch discrimination may be of crucial importance for well-trained violin players, whereas such automatical detection of pitch differences is not available for nonmusicians.

Tervaniemi et al. (2005) have further argued that the differences between musicians and nonmusicians in pitch change processing result from the effects of parametric manipulations on the magnitudes of pitch deviance and the subjects' attentional focus [60]. To confirm the specificity of pitch discrimination accuracy in musicians, all the participating musicians in their study reached professional levels in different instruments (i.e., guitar, wind, piano, and string instruments). The authors utilized small pitch differences in relation to threshold to provide evidence that musicians have smaller, just noticeable differences in pitch perception than nonmusicians. Meanwhile, large pitch differences were employed to examine whether and how musicians' superiority is reflected in behavioral and ERP measures at suprathreshold levels. Partially consistent with the conclusion by Koelsch et al. (1999), the behavioral data in Tervaniemi et al. (2005) have revealed that the superiority of pitch discrimination accuracy in musicians over nonmusicians was observed not only with the 0.8% (small pitch differences) but also with the 2% (large pitch differences) frequency changes. Here, we need to explain briefly that there were 3 kinds of deviant sounds (0.8%, 2%, or 4% changes in frequency), and the so-called “preattentive” (0.8%) and “attentive” (2% or 4%) level were defined by the just noticeable differences in pitch perception. Furthermore, nonmusicians could also detect quite reliable and small pitch changes of 0.8%. However, the ERP data, that is, the MMN and the P3a, did not differentiate between nonmusicians and musicians in both two levels of pitch differences. These results suggest that musical expertise may exert its effects merely at attentive levels but is not necessary at the preattentive levels.

*(2) Interval Made by Pitches*. Besides pitch perception, MMN is also affected by some specific interval structure changes in music, that is, the distance between two serial pitches or several simultaneous pitches. In an ERP study by Brattico et al. (2003), they revealed that MMN showed a larger amplitude to an infrequent dissonant interval (the major seventh) in a context of repeated consonant intervals (the major sixth) than to an infrequent consonant interval in a consonant context (e.g., the perfect octave replacing the major sixth) [61]. Importantly, the similar result was obtained even if the pitch deviation between consonant standards and dissonant deviants was smaller in pitch distance than that between consonant deviants and consonant standards. This result may reject the assumption that MMN increases its amplitude with acoustically larger pitch shifts (e.g., Tiitinen et al. [62]). Accordingly, Tervaniemi and Brattico (2005) have demonstrated that human brain responds more vigorously to the distance in dissonance between infrequent musical interval and the repeated context than to the distance in pitch between their tone components.

*(3) Contour: A Kind of Sound Context*. Several studies have suggested that contour may be a more fundamental attribute for melody recognition than interval size [63]. It is a defining feature of melodies and hence is a potent cue to the identity of musical pieces, such as short note sequences that form recognizable “hooks” and “themes.” Ample evidence has proved that contour processing provides an essential basis for melody recognition [64–66]. For example, Dowling [67] has investigated the role of both interval and contour in melody recognition. In the interval condition, subjects were asked to detect subtle deviations in interval size between referential and target melodies that were played in either the same or different keys. The results showed that subjects yielded more errors when the target melodies were presented in different keys than when those were presented in the original key. In the contour condition, subjects were instructed to detect deviations in the contours of target melodies that were played in either the same or different keys, and subjects' performance was robust across transpositions between different keys

Contour also provides a kind of musical sound context in MMN paradigms. Brattico et al. (2001) have compared musicians with nonmusicians on their accuracy of processing pitch deviations of identical magnitude in temporally complex auditory contexts (Western versus non-Western scales) and in single sounds [68]. The results showed that the pitch deviation in the Western condition evoked larger MMN amplitude than that in the non-Western condition and in the single-tone condition. This suggests that cognitive processing of pitch deviation between the subsequent tones is facilitated in a complex sound context with familiar frequency ratios (Western condition) compared to unfamiliar frequency ratios (non-Western condition) and single tones. In addition, violations of music scale rules in unfamiliar melodies also elicit an MMN-like response with main generators localized in the nonprimary auditory cortex [38]. All these findings suggest that the music MMN reflects violations of musical regularities that are stored in long-term memory, rather than that developed during the short-term memory formation.

#### 2.4.2. Attentive or Preattentive of Beat Processing?

Beat is perceived regularly as salient events in time sequence by listeners. And this has been suggested to be domain-specific as well as species-specific to human beings [69]. Although some previous studies have reported that rhythmic entrainment may be a byproduct of the vocal learning mechanisms that are shared by some kinds of animals, including human beings [70, 71], Honing et al. (2012) have used the oddball paradigm to compare different abilities to detect the deviant musical features between human beings and rhesus monkeys (*Macaca mulatta*) and revealed that human beings detected the beat in music, but the rhesus monkeys did not [72].

Then the beat perception in human beings is an attentive or a preattentive processing? Some researchers have suggested that focused attention is generally necessary for both beat perception [64, 65] and regularity detection [66]. In contrast, others have evidenced that beat processing is in fact preattentive [73], since it runs in sleeping newborns [74]. In the original beat-processing paradigm, researchers manipulated temporal structures of rhythm as metrical structures [64] and highly syncopated rhythms [65]. Ladinig et al. (2009) later controlled metrical stimuli more strictly by adding the variation in the timbre and intensity of tones to convey the metrical structure [73]. And thus, it is more acoustically rich and ecologically valid, allowing listeners to detect a beat preattentively.

However, a question arises here: whether or not the different responses to tones in different metrical positions are due to acoustic differences rather than beat processing? To address this issue, Bouwer et al. (2014) have used an MMN paradigm to examine the beat processing [75]. In their study, both musicians and nonmusicians were presented with a varying rhythm with a clear accent structure, in which sound was omitted occasionally. They compared MMN responses to the omissions of identical sounds in different metrical positions. The result showed that omissions in strong metrical positions (on the beat) elicited higher amplitude MMN responses than those in weak positions (not on the beat). This suggests that detection of a beat is preattentive when highly beat inducing stimuli are used. Importantly, they did not find any effect of musical expertise, suggesting that beat processing in metrically simple rhythms with clear accents does not need attention or musical expertise. Therefore, we indeed need to pay much attention to the fact that how acoustically varying stimuli may influence ERP results in further studies.

#### 2.4.3. MMN in Single Chord Manipulation

Western music has two classifications that are familiar to all Western listeners: dichotomy of minor and major modalities and that of consonance and dissonance. By changing the interval structure of a chord, there exist some chord MMN paradigms, such as discrimination of slightly mistuned chords [54, 63]. However, in previous MMN studies that used chords as standard and deviant stimuli, the deviant chord included tones that were not present in the standard chord. Consequently, one possibility is that MMN could be simply elicited by the infrequently occurred tones, without necessarily reflecting any higher-order, musical differences (minor versus major) between the standard and deviant chords.

To resolve this problem, Virtala et al. (2011) have examined processing of chords in a new paradigm, where different standard chords were presented [76]. The deviant chords consisted of the same notes as in the standards but were different in qualitative combinations. They used all possible root major triads as standard stimuli. In order to assess discrimination of both the major-minor and consonance-dissonance categories, there were 3 kinds of deviant stimuli: minor chords, dissonant chords, and inverted major chords (each was represented by three different, equiprobable chords from that category). In addition, to ensure elicitation of a classical physical-feature MMN, their sequences also included occasional standard major chords but are slightly softer in loudness. In the ignorance condition, the MMN was significantly elicited by all types of chords, but not by the inverted major chords. In the detection condition, the MMN was significantly elicited by the dissonant chords and the soft target chords. However, whether the classifications of major versus minor modalities and consonance versus dissonance are innate or based on implicit or explicit learning remain a question for future studies.

#### 2.4.4. Timbre Difference in MMN

Many researchers do agree that, compared with chord processing, different instrumental tones (timbre) are processed by a different cortical area [77], and the stimulus complexity might influence the processing of musical sounds. Using a novel, fast multifeature MMN paradigm, Vuust et al. (2012) have investigated preattentive processing of auditory features in musicians with expertise in four distinct styles of music (classical, jazz, rock, or pop) and in nonmusicians [78]. Their results revealed that jazz musicians showed the greatest auditory expertise effect, especially when compared with rock musicians and nonmusicians. One possible interpretation is that jazz is characterized by complex harmonies and elaborate melodic and harmonic materials that present a challenge to many listeners. Furthermore, since jazz is often improvised, the variations of the harmonic progressions and rhythmic feelings are more frequently communicated across jazz musicians [79]. Thus, specific auditory skills, required for performing different musical tasks such as conducting an orchestra [80, 81], playing certain instruments or musical genres, lead to sensitivity to specific sound features, inducing different MMN responses in the amplitude and the latency (for a review, see [82]).

## 3. Neural Plasticity in Music MMN

### 3.1. Do Musicians Show More MMN Effects?

Musicians have various pronounced skills in auditory perception that correlate with their expertise in music. For instance, musicians can detect smaller pitch differences, but nonmusicians cannot. In addition, musicians show better performance in discriminating pitch intervals [83] and in structuring rhythms [84] than nonmusicians. And these superior auditory processing skills of musicians are reflected in the MMN. For example, chords that deviated from repetitive standard chords by only 0.75% in pitch are enough to elicit MMN in violinists, whereas a much larger pitch deviation is required to elicit MMN in musical novices.

#### 3.1.1. Different Time Grouping

Musicians also have a longer time window for integrating sounds as indicated by MMN elicitation [85]. van Zuijen et al. (2005) have showed that the auditory system of musicians organize sounds differently from that of nonmusicians [86]. Furthermore, professionally trained classical musicians showed auditory grouping of a tone sequence into four-tone groups according to a more complex Gestalt-grouping rule than nonmusicians. The authors found evidence on auditory grouping according to pitch-similarity in both musicians and nonmusicians. According to a good-continuation-of-pitch, however, the grouping was only observed in musicians. Grouping of the sequence was indicated by MMN response to an occasional fifth tone that extended the length of the standard-tone groups. The deviant could therefore have been detected by encoding a temporal and/or numerical regularity.

Furthermore, van Zuijen et al. (2005) have examined whether auditory system could encode a temporal regularity or a numerical regularity and the differences between musicians and nonmusicians. To achieve this goal, van Zuijen et al. presented tone sequences containing either a temporal regularity or a numerical regularity to participants. The sequence with the temporal regularity could be divided into segments with a constant duration but contained a varying number of tones. And the sequence with the numerical regularity could be divided into segments containing a constant number of tones but varied in duration. Auditory encoding of the regularity was then determined by examining whether MMN was elicited by an occasional segment lengthening varied in time (duration) or in number. The results revealed that, in both musicians and nonmusicians, an MMN was elicited when the temporal regularity was unexpected. In contrast, an MMN was elicited to violations of the numerical regularity only in musicians. These results suggest that temporal processing is important in audition, since a complex temporal regularity can be encoded at an involuntary auditory processing stage regardless of musical expertise. Furthermore, the auditory system of professional musicians can encode a numerical regularity without attention, reflecting the functional importance of beat tracking in the perceptual organization of music.

#### 3.1.2. What Factors Shaped Musician's Expertise?

Musicians' brains are shaped by their training types, musical styles or genres, and listening experiences. Accordingly, their neural activations depend highly on the instruments they played and the practice strategies, as well as the levels of their expertise. For instance, musicians who need to intone while playing such as violinists normally show a greater sensitivity to small deviations in pitch than musicians playing other instruments and nonmusicians [78]. And singers also yield a stronger MMN response than instrumentalists to small pitch deviations [87]. In addition, rhythmic deviations elicited a stronger, faster, and more left-lateralized MMN in musicians than in nonmusicians [88]. More recently, it is reported that musicians who perform music primarily without a score learn more easily to detect contour deviations in melodic patterns than those who read music score while playing, and this difference is detected preattentively by an enhanced MMN response [89]. Taken together, these studies indicate that musicians' brains process auditory information differently according to their practice strategies, and the MMN is sensitive to acoustic features that are specific to musical objects or practice.

#### 3.1.3. Different Speed and Magnitude of Neuroplasticity

The MMN is modulated by musicianship as well [85, 86]. For instance, increased ERPs are measured in musicians especially when the sounds are complex [85]. Consistently, in Seppänen's study (2013), musicians also showed enhanced auditory processing compared with nonmusicians [90]. They examined the neural bases of the musical training effects on rapid plasticity in auditory processing. The learning-related plastic changes in ERP responses to pitch and duration deviants between passive blocks were compared between musicians and nonmusicians. Passive blocks were interleaved with an active discrimination task, in which participants were instructed to focus on an unrelated task but to ignore the testing task. In contrast, in the active blocks participants were asked to pay their attention on both tasks. The results showed that deviant-related ERP responses around the parietal areas decreased after finishing the active task in both musicians and nonmusicians. Compared with nonmusicians, musicians showed larger MMN responses, especially to deviations in musical sounds such as chords [54], melodies [51], and rhythms [56] as well as other complex auditory stimuli [78, 91].

#### 3.1.4. Differences of Brain Structure

Previous studies have revealed increased gray matter volume and density in auditory cortices of musicians [92, 93]. On the basis of the structural findings, changes in the auditory ERPs in musicians may indicate expanded activation areas, increased number of neurons, greater synchronization, or faster connectivity. For instance, the results of James et al. (2013) revealed rapid plastic changes in the bilateral temporal and the left frontal areas only in musicians, but not in nonmusicians [94].

### 3.2. How Do the MMN Effects Change over Time by Natural Exposure?

It seems that human beings are born for detection of sound discriminations. The MMN responses to auditory stimuli have been successfully recorded even in fetus and newborns or preterm infants [95]. Previous research has mainly focused on five developmental periods: fetus, inborn, preschool children, adults, and aging individuals ([96], for a review, see [97]), and suggests that MMN is developmentally stable and has a unique U-shaped curve. The MMN amplitude is only slightly smaller in infants than school-age children, and there is no MMN difference between school-age children and adults. With respect to the MMN latency, it is slightly longer in infants than in adults but reaches adult level quickly by early school-age years. Child MMN, however, does not seem to be analogous to adult MMN. For example, compared with adult, a prominent MMN response could be obtained in both waking and sleep states in infants. Furthermore, the distribution of MMN scalp is relatively broader and more central in children than in adults (for a review, see [98]).

Concerning music conception, previous behavioral studies have demonstrated that, at around the age of six months, infants are already equipped with certain perceptual and cognitive prerequisites for putative beneficial effects of immersing in a musical enriched environment. They not only show fairly accurate discrimination of basic musical features such as pitch and duration but also are sensitive to some abstract aspects of musical sounds. For example, infants, encoding melodies and rhythms depending on features of relative pitch and duration, are able to group individual tones by pitch and show long-term memory for musical pieces. More recently, ERP studies have shown that music-relevant auditory abilities, such as discrimination of different intervals, sound grouping, perception of missing fundamental, auditory stream segregation, and detection of the beat of rhythmic sounds, are available before the age of six months or even at birth.

These early perceptual skills are also important in childhood: young children who typically receive ample musical exposure tend to find music both interesting and enjoyable. Thus, everyday musical activities are rich sources of experience that may shape the development of auditory skills. In an ERP study, Trainor et al. (2011) randomly assigned 4-month-old infants either to a guitar group, in which they were exposed to recordings of melodies in a guitar timbre, or to a marimba group, in which they were presented with the same melodies in a marimba timbre [99]. After a week-long, 20 min per day exposure to one of the two timbres, the guitar-exposed infants showed a larger obligatory response to guitar tones than to marimba tones, and the opposite response pattern was found for the marimba-exposed group. Furthermore, occasional pitch deviations in guitar tones elicited an MMN only in the guitar-exposed infants, whereas pitch deviations in the marimba tones elicited a significant MMN in both groups. These results suggested that a relatively short-term exposure to music in infants is enough to strengthen the neural representations of a given timbre, which is further reflected by MMN response to that timbre pitch.

### 3.3. Could MMN Effects Be Trained?

Recently, growing studies have provided evidence on a modulation role of training (or experience) in MMN effects. For instance, François et al. (2012) have demonstrated that 8–10-year-old children who had been randomly assigned to (12 months) a music lesson group showed a larger increase in MMN amplitude than the control group children who were assigned to a painting lesson [100]. Consistently, Putkinen and Saarikivi (2013) have also revealed that the MMN elicited by minor chord deviants in a sequence of major chord standards showed more amplitude increase in music-trained children (7–13 years old) than in children without music training [101].

It is noteworthy that the children participants in music studies all received formal training of music. However, in our daily life only a few people have opportunities to obtain professional musical trainings. Most of normal people are nonmusicians but amateurs or just influenced by musical contexts in an implicit way. For instance, various computer and console games attract children into “musical play,” which could be considered as informal environments for music learning [101]. Then, an important question is that do these informal musical activities affect development of auditory discrimination and attention in preschool-aged children? This has been confirmed at least in adult participants. In previous behavioral studies, adults who have no experience of musical training showed (implicit) competence in processing of some fairly nuanced aspects of music, as a similar manner of learning through mere incidental exposure.

Previous ERP studies have demonstrated that brains of nonmusicians process some aspects of Western tonality and harmony automatically [102]. One assumption is that these idiosyncrasies of Western tonal music are internalized in nonmusicians via everyday musical experiences. Recently, Putkinen and Saarikivi (2013) have investigated an issue of whether amount of informal musical activities is related to electrophysiological correlates of auditory deviation detection in 2-3-year-old children [101]. They used a multifeature MMN paradigm (Näätänen et al., 2004) to assess several auditory ERP responses and revealed that the MMN reflects different stages of auditory processing particularly in childhood. This result suggests that ambient exposure to musical contexts without special training is sufficient for learning of culture-specific musical knowledge. More importantly, one question rises as whether informal exposure to music may also modulate development of auditory processing beyond musical domain.

## 4. Conclusions and Further Directions

There is no doubt that MMN has opened a window for better understanding of music perception from a perspective of cognitive neuroscience. The MMN paradigms have been gradually evoking to get close to the real music. And consequently, it has become an effective index of perception of regularities in music. By now, almost all neuroscience studies on music had found the MMN features in basic levels of music, such as pitches, timbres, intensities, and short-term melodies. And the temporal processing in MMN is similar among these basic musical elements. That is, the MMN normally peaks at around 100–200 ms after deviation onset, and its amplitude and latency alter depending on deviation magnitude and related perceptual discriminability, which may be modulated by expertise effects and musical sound contexts. Concerning the neural basis of MMN, we still cannot reach a common idea about the exact position of the MMN's main source in brain due to nonuniform musical materials and paradigms. It is certain that, however, our brain is inborn to detect MMN features in music, and this ability changes over time in U-shaped route [103].

On the other hand, MMN has shown its feature of plasticity (i.e., neuroplasticity). For instance, different MMN responses have been consistently revealed between musicians and nonmusicians. And these differences mainly resulted from training efforts, which include natural exposure to music contexts and informal musical trainings. Because of its features of deviation-sensitivity and neuroplasticity, MMN has also functioned well in some clinical and practical areas, such as assessment of the wholeness of central auditory system in infants, detection of innate predisposition for musical preferences, and treatment of musical abilities of adult cochlear implant users. Although we have made great achievements in music MMN, there still exist many open questions needed to be resolved in future studies.

### 4.1. MMN and Music Syntax

The relationship between serial and hierarchical processing has been discussed in language literature [104]. Little study, however, has addressed this issue in music perception. Since music has complicated structures and regularities, perception of music involves various cognitive functions. In contrast, MMN is one indicator of brain responses to music and reflects primarily low level of music elements. The higher hierarchical structures of music, such as chord progressions (harmonic progressions), may be also a key component of music perception and are out of reach of MMN. Previous research has suggested that there is no single way to fully understand syntax in music, but combination of MMN and an early right-anterior negativity (ERAN) may create a new direction of this area. Both ERAN and MMN are event-related potentials elicited by detection of deviations of regularities and have similar peak amplitude and latency. MMN mainly detects abstract-feature deviants (frequency, intensity, location, gap, etc.), while ERAN is an ERP response to harmonically inappropriate chords when listening to music. That is, MMN representations of regularities of intersound relationships are extracted online from acoustic environments, whereas ERAN represents music-syntactic regularities that already exist in long-term memory, which have been shaped by different musical cultures (for a review, see [105]). Taken together, to fully understand the neural bases of music perception, in the current stage, it is necessary to combine ERAN and MMN in music neuroscience research. However, the relationship between ERAN and MMN needs to be examined further.

### 4.2. MMN from a Developmental Perspective

Previous studies have suggested that we process MMN features congenitally. And this has been evidenced by several research results, such as frequency and intensity discrimination in fetus and infants [95]. To confirm this assumption, however, it is essential to examine more acoustic features, music features in particular. The neuroscience studies on real music features enable us to better understand a series of important issues on music perception/apprehension; for instance, whether or not human beings have an innate ability to distinguish musical regularities by MMN, and which MMN features are flexible to be influenced via training? When do MMN differences between musicians and nonmusicians begin to develop? And whether or not significant brain structural changes induced by early training were sustained even if musical training ceased thereafter?

Research outside of music domain in healthy adults has reported that brain structural changes that resulted from a complex motor task (e.g., juggling) normally occur within 1 week of training onset but return to baseline without ongoing training. In field of music neuroscience, however, no study has focused on these points. And two main issues needed to be considered in future studies are that “is there any ‘optimal period' that maximizes difference during music training” and “whether or not levels of musical expertise can slow down attenuation of MMN effects”?

### 4.3. Wide Applicability of Musical MMN

MMN is well accepted for its features of being noninvasive and having no need for explicit awareness (preattentive) and hence has a wide range of applications in research and clinical areas. For example, MMN recorded in an unconscious coma patient provides reliable predictor of recovery of consciousness. With respect to MMN in sleep, it has weak effect in adults, but in infants and newborns MMN is very helpful in assessing their central auditory functions. It is worthy to note that, besides healthy individuals, MMN paradigm is also important for individuals with specific diseases or disabilities, such as cochlear implant (CI) users and individuals with schizophrenia. Specifically, for the CI users, MMN paradigm is not only an evaluation criterion to the quality of implantation, but also a good investigation tool to assess residual neural functions of processing musical sound features or inborn musical abilities [98]. However, this still exists as an academic discussion now, and it is necessary to develop related products following rationale of MMN in near future.

### 4.4. Studying Music in a More Musical Context

Music is a fine-grained aesthetic entity. When presented with simple sinusoidal tones with greater mistuning instead of fine-grained differences, violinists were not superior to nonmusicians in discriminating pitch deviations. A similar lack of MMN differences was also obtained when comparing processing of isolated infrequent sinusoidal tones or infrequent minor chords within a sequence of major chords between musicians and nonmusicians [38]. Seppänen et al. (2007) have compared musicians who mainly employ auditory rehearsal and playing strategies to a nonaural group as determined by a questionnaire. They found that practice strategies modulate the speed of neural discrimination of interval and contour deviations presented in melody-like patterns [89], but not in simple sound features. Although so-called “perfect melodic multi-feature MMN paradigm” [28] has showed much better ecological effects than other paradigms, it is just music-like. Music has various styles, consisting of different genres and composing ways, and thus it will be hard for researchers to develop a unified, standard paradigm that represents all acoustic features. Therefore, it is necessary to classify music styles first and then design different music paradigms correspondingly.

### 4.5. Daily Interactions with Music

Previous music MMN studies have been primarily conducted in well-controlled laboratory environments and in turn lack ecological effects leading to distinctive results in different musical contexts. One possibility is that different participants may have different levels of interactions with music in their daily life [102]. Some researchers have investigated several indices of musical experiences of participating children from their parents, such as how often the children engaged in different types of musical activities at home (e.g., singing and dancing) and how often they played music with their children (e.g., how often they sang to their children or sang with their children together). And a composite score representing the amount of such musical activities in daily life was significantly correlated with the ERP response amplitudes, such as enlarged P3a responses to duration and gap deviants and a diminished LDN (late discriminative negativity) across all five deviant types.

### 4.6. Enculture

From a perspective of society, few people will deny that music is a universal language across different cultures. However, music still does not reach a common consensus cross-culturally in cognitive neuroscience level. Previous studies have consistently demonstrated that differences in cultural backgrounds affect the way in which people perceive auditory signals [106]. For instance, subjects from tonal language cultures (e.g., Chinese) performed better than those from nontonal language cultures (e.g., French) in pitch discrimination [107]. Accordingly, various open questions, such as whether there is an early sensitive period for the culture effects, whether the culture effects are relatively stable or malleable by musical experience, and whether the children who experience different musical cultures have the same level of sensitivities to musical MMN, are all interesting topics.

On the other hand, previous research of music neuroscience has mainly focused on Western music system (i.e., diatonic scale or equal temperament). Little is known, however, about the MMN in non-Western music (i.e., pentatonic scale). Therefore, we also need to pay much attention to non-Western music in further studies, such as ancient Chinese music, Indic music, and Japanese music. That is, the similarities and differences of processing of music perception cross-cultures are needed to be examined via intercultural comparison for better understanding of neural mechanisms of music perception/apprehension and music per se.
